# Recombinant Vectors Based on Porcine Adeno-Associated Viral Serotypes Transduce the Murine and Pig Retina

**DOI:** 10.1371/journal.pone.0059025

**Published:** 2013-03-08

**Authors:** Agostina Puppo, Alexander Bello, Anna Manfredi, Giulia Cesi, Elena Marrocco, Michele Della Corte, Settimio Rossi, Massimo Giunti, Maria Laura Bacci, Francesca Simonelli, Enrico Maria Surace, Gary P. Kobinger, Alberto Auricchio

**Affiliations:** 1 Telethon Institute of Genetics and Medicine, Napoli, Italy; 2 Medical Genetics, Department of Medical Translational Sciences, University of Napoli Federico II, Napoli, Italy; 3 Special Pathogens Program, Public Health Agency of Canada, National Microbiology Laboratory, Winnipeg, Manitoba, Canada; 4 Department of Medical Microbiology, University of Manitoba, Winnipeg, Manitoba, Canada; 5 Department of Ophthalmology, Second University of Napoli, Napoli, Italy; 6 Department of Veterinary Medical Science, University of Bologna, Ozzano dell'Emilia, Bologna, Italy; University of Kansas Medical Center, United States of America

## Abstract

Recombinant adeno-associated viral (AAV) vectors are known to safely and efficiently transduce the retina. Among the various AAV serotypes available, AAV2/5 and 2/8 are the most effective for gene transfer to photoreceptors (PR), which are the most relevant targets for gene therapy of inherited retinal degenerations. However, the search for novel AAV serotypes with improved PR transduction is ongoing. In this work we tested vectors derived from five AAV serotypes isolated from porcine tissues (referred to as porcine AAVs, four of which are newly identified) for their ability to transduce both the murine and the cone-enriched pig retina. Porcine AAV vectors expressing EGFP under the control of the CMV promoter were injected subretinally either in C57BL/6 mice or Large White pigs. The resulting retinal tropism was analyzed one month later on histological sections, while levels of PR transduction were assessed by Western blot. Our results show that all porcine AAV transduce murine and porcine retinal pigment epithelium and PR upon subretinal administration. AAV2/po1 and 2/po5 are the most efficient porcine AAVs for murine PR transduction and exhibit the strongest tropism for pig cone PR. The levels of PR transduction obtained with AAV2/po1 and 2/po5 are similar, albeit not superior, to those obtained with AAV2/5 and AAV2/8, which evinces AAV2/po1 and 2/po5 to be promising vectors for retinal gene therapy.

## Introduction

Gene therapy with recombinant adeno-associated viral (AAV) vectors is emerging as a promising therapeutic strategy for inherited retinal degenerations (IRDs) [1], a group of blinding conditions including retinitis pigmentosa [2] and Leber congenital amaurosis (LCA) [3], for which no treatment is currently available. The results from three independent clinical trials in patients with LCA type 2 (LCA2) due to mutations in RPE65 show that subretinal administration of AAV serotype 2 vectors is safe and effective [4]–[14]. These data bode well for future translation of AAV-mediated retinal gene transfer to other IRDs, especially those like LCA2 that require gene transfer to the retinal pigment epithelium (RPE).

Gene transfer to photoreceptor cells (PR) in the retina appears more challenging than to RPE, possibly due to the presence of the interphotoreceptor matrix and the outer limiting membrane, which may limit access to PR bodies from the subretinal space where the vector is injected [15]–[16]. However, efficient gene transfer to PR is essential for the treatment of IRDs as the majority of genes involved in Mendelian forms of IRDs are expressed in PR [17]. There are over one hundred different AAV isolates/serotypes available [1]. These can be converted to a similar number of AAV vectors, each containing the genome of AAV2 and an heterologous capsid from a different serotype (referred to as AAV2/n, where the first number defines the genome and the second the capsid of origin included in the AAV vector) thus offering a unique opportunity to select the best AAV serotype for PR gene transfer. Our group has shown that AAV2/5 transduces efficiently PR upon subretinal administration [18]. In an ongoing search for more efficient AAV serotypes for PR transduction, we then demonstrated that AAV2/8 outperforms AAV2/5 in transducing PR of mice [19] and pigs [20]. Other groups have shown the potential of both AAV2/5 and AAV2/8 in transducing non-human primate (NHP) PR [21]–[22] and rescuing animal models of IRDs [23]–[24]. However, the search for AAVs that are more efficient than existing vectors in transducing the retina is prompted by the continuous identification of novel AAV serotypes.

Recently, AAV have been derived from AAV sequences isolated from porcine tissues [25]. These vectors appear to be promising for transduction of a variety of tissues, including the retina [25]. Here we report the isolation of capsid sequences from 4 novel AAVs from porcine tissues, and the generation of the corresponding AAV vectors. The retinal transduction abilities of these as well as the previously described porcine AAV2/po1 were compared to those of AAV2/5 and 2/8 in mice and pigs (which have large and cone-enriched retinas [26]) in order to test the tropism of the viral vectors.

## Materials and Methods

### Isolation and Cloning of Porcine AAV Sequences

Porcine genomic DNA was isolated as described below [25]. Briefly, porcine genomic DNA was isolated from pig gut using a QIAamp DNA Mini kit (Qiagen, Valencia, CA, USA). Porcine genomic DNA was screened for the presence of AAV using primers RC^+^: 5′-GGTGCGTAAACTGGACCAATGAGAAC-3′ and SIG^-^: 5′-GAATCCCCAGTTGTTGTTGATGAGTC-3′, isolating an ∼1.7 kb region spanning the end of *rep* and beginning of *cap*. The DNA fragments were sequenced by the DNA Core Facility at the National Microbiology Laboratory (NML) and BLAST analysis was performed to determine whether the fragments were indeed unique. It was observed that several conserved regions in *rep* were present in AAV isolates from pig genomic DNA, and these regions were used to design three specific consecutive primers meant to isolate full-length *cap* in a nested thermal asymmetric interlaced polymerase chain reaction (nested TAIL PCR), along with a degenerate primer. The three successive primers were Nestedcap1^+^2.7 kb, Nestedcap2^+^2.4 kb, and Nestedcap3^+^2.2 kb (5′-TGGAGGACCGAATGTTCAAGTTTG-3′, 5′-CGACCGATCGATTGGACCTC-3′, and 5′-AAGCGAGTAGTCATGTCGTTG-3′, respectively). The degenerate primer, as previously described, was CED^-^ (5′-ACTGAMACGAAT(H/-)AMMCGGTTTATTGA-3′) [25]. PCR products were purified using a QIAquick Gel Extraction kit (Qiagen) and cloned into pCR2.1-TOPO Vector (Invitrogen, Carlsbad, CA, USA). With this method, *cap* for AAVpo4, -po5, and -po6 were obtained (GenBank accession: JX896667, JX896666, and JX896664, respectively). The number given to each porcine AAV serotype corresponds to the order in which they were identified. Previously, only a partial sequence for AAVpo2 had been isolated. Since AAVpo2 shares high homology with AAVpo6, we were able to create a hybrid, which we called AAVpo2.1, using the beginning portion of AAVpo6 to complete the *cap* for AAVpo2 (GenBank accession JX896665). AAVpo1 was isolated as described above [25]. Once the full-length AAV *cap* was obtained from pig genomic DNA, the *cap* was cloned into p600 using SwaI and NotI restriction sites [25], thus generating pACK2/po1, 2/po2.1, 2/po4, 2/po5, and 2/po6.

**Figure 1:**
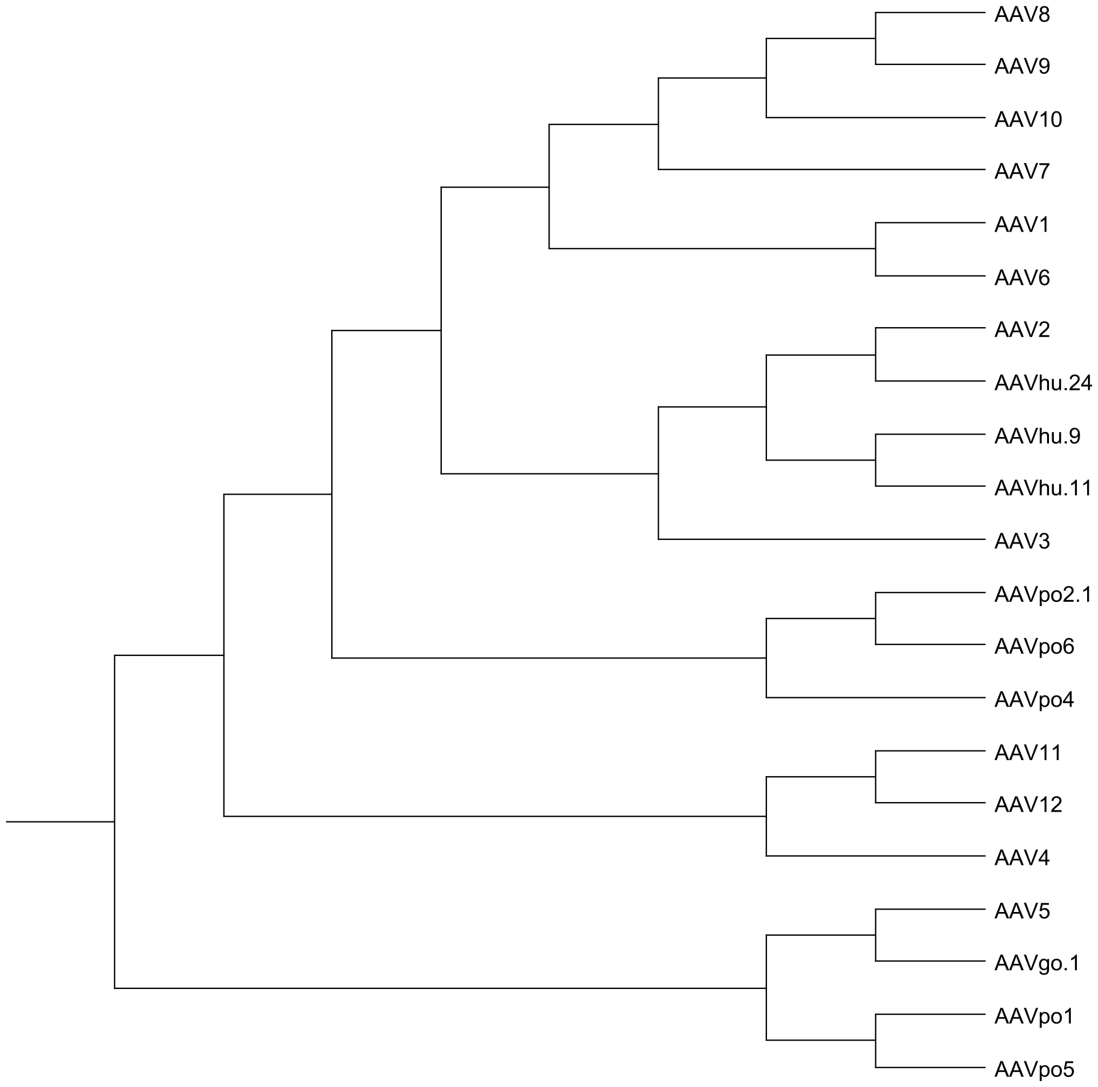
Phylogenetic tree obtained through the alignment of the *cap* sequences of the newly isolated porcine serotypes and other preexisting AAVs.

In Figure 1 the *cap* amino-acid sequence from AAVpo1, -po2.1, -po4, -po5, and -po6 were aligned with various AAVs representing each clade using the Clustal W Method of DNASTAR MegAlign software (DNASTAR, Madison, Wisconsin, USA).

### AAV Vectors Production and Purification

AAV vectors were produced by the TIGEM AAV Vector Core (Napoli, Italy) using the pAAV2.1-CMV-EGFP [27] and -Rho-EGFP [19] expression plasmids, and the packaging pACK2/po1, 2/po2.1, 2/po4, 2/po5, and 2/po6 plasmids (described above). Recombinant AAV2/5, 2/8, 2/po1, 2/po2.1, 2/po4, 2/po5 and 2/po6 vectors were produced through the triple transfection of 293 cells followed by cesium chloride purification [18]. For each viral preparation, titers (in GC/milliliter) were determined by both PCR quantification using TaqMan (PerkinElmer Life and Analytical Sciences, Boston, MA, USA) and dot-blot analysis [28].

### Animal Procedures and Vector Administration

#### Ethics Statement

All studies on mice were conducted in strict accordance with the institutional guidelines for animal research and with the Association for Research in Vision and Ophthalmology (ARVO) Statement for the Use of Animal in Ophthalmic and Vision Research. All procedures on mice were submitted to the Italian Ministry of Health; Department of Public Health, Animal Health, Nutrition and Food Safety on October 17th, 2011. The Ministry of Health approved the procedures by silence/consent, as per article 7 of the 116/92 Ministerial Decree.

All experiments involving pigs were conducted according to relevant national and international guidelines. All procedures on pigs were reviewed and approved in advance by the Scientific Ethics Committee for Animal Experimentation of the University of Bologna (Bologna, Italy) and were then approved by the Italian Ministry of Health (protocol number: 23/2009-B, approval date Feb. 04, 2009). All surgery was performed under anesthesia, and all efforts were made to minimize suffering.

#### Mice

Four-week-old C57BL/6 mice (Harlan, S. Pietro al Natisone, Italy) were anesthetized with an intraperitoneal injection of avertin (1.25% w/v of 2,2,2-tribromoethanol and 2.5% v/v of 2-methyl-2-Butanol; Sigma–Aldrich, St. Louis, MO) at 2 ml/100 g of body weight. A volume of 1 µl of viral vectors was delivered subretinally or intravitreally via a trans-scleral transchoroidal approach, as previously described [29]. Following injection, the extent of transduction was assessed by ophthalmoscopy one month post-injection, and the eyes were then harvested.

#### Pigs

Eleven-week-old Large White (LW) female piglets were utilized. Pigs were fasted overnight leaving water *ad libitum*. The anesthetic and surgical procedures for pigs were previously described [20].

AAV vectors were inoculated subretinally in the avascular nasal area of the posterior pole between the two main vascular arches, as performed in Mussolino et al [20]. This retinal region is crossed by a streak-like region that extends from the nasal to the temporal edge parallel to the horizontal meridian, where cone density is high, reaching 20000 to 35000 cone cells mm^2^ [26]. Each viral vector was injected in a total volume of 100 µl, resulting in the formation of a subretinal bleb with a typical ‘dome-shaped’ retinal detachment, with a size corresponding to 5 optical discs.

### Histological Analysis

Mice were sacrificed and their eyeballs were harvested and fixed overnight by immersion in 4% paraformaldehyde (PFA). Before harvest the temporal aspect of the sclerae was marked by cautery to orient the eyes with respect to the injection site at the moment of the inclusion. The eyeballs were cut so that the lens and vitreous could be removed leaving the eyecup intact. Mice eyecups were infiltrated with 30% sucrose for cryopreservation and embedded in tissue freezing medium (O.C.T. matrix, Kaltek, Padua, Italy). For each eye, 150 to 200 serial sections (10 µm-thick) were cut along the horizontal plane and the sections were progressively distributed on 10 slides so that each slide contained 15 to 20 sections, each section representing the whole eye at different levels. The sections were stained with 4′,6′-diamidino-2-phenylindole (Vectashield, Vector Lab Inc., Peterborough, UK) and EGFP was monitored with a Zeiss Axiocam (Carl Zeiss, Oberkochen, Germany) at different magnifications.

Pig eyeballs were fixed overnight by immersion in 4% PFA. The eyeballs were cut so that the lens and vitreous could be removed, leaving the eyecups in place. The eyecups were cryoprotected by progressive infiltration with 10%, 20%, and 30% sucrose. Before embedding, the swine eyecups were analyzed with a Fluorescence stereomicroscope (Leica Microsystems GmbH, Wetzlar, Germany) in order to localize the transduced region. Embedding was performed in tissue-freezing medium (O.C.T. matrix, Kaltek, Padua, Italy). For each eye, 200 to 300 serial sections (12 µm- thick) were cut along the horizontal meridian and the sections were progressively distributed on glass slides so that each slide contained 6 to 10 sections. Sections were stained and image acquisition was performed using a Leica microscope (Leica Application Suite v. 2.3.6 DM6000B, Leica Microsystems).

### Hematoxylin and Eosin Staining

For hematoxylin and eosin staining, tissues were washed with sodium phosphate buffer (PBS) for 3 minutes. Next, the slides were stained with hematoxylin 10% (Sigma-Aldrich, Milan, Italy) for 3 minutes, rinsed with deionized water, and stained with eosin (Sigma-Aldrich, Milan, Italy) for 1 minute. Tissues were dehydrated in a series of alcohol dehydration steps (80%, 90%, 95% and 100% ethanol), mounted with xylene-based mounting media and covered with a coverslip. The retinal histology was then analyzed by light microscopy.

### Fundus Photography

Fundus photographs of mice were taken with a Topcon TRC-50IX retinal camera connected to a charge-coupled-device Nikon D1H digital camera (Topcon Medical System). Mice ocular fundus, after dilating the pupils with a drop of tropicamide 1% (Visufarma, Roma, Italy), were photographed using a 300 W flash.

### Western Blot Analyses for EGFP Quantification

Western blot analysis was performed on retinas, which were harvested as described above [30]. Samples were lysed in hypotonic buffer (10 mM Tris-HCl [pH 7.5], 10 mM NaCl, 1,5 mM MgCl2, 1% CHAPS, 1 mM PMSF, and protease inhibitors) and 50 µg of these lysates were separated by 12% SDS-PAGE. After the blots were obtained, specific proteins were labeled with anti-EGFP (1∶500; Santa Cruz Biotechnology, sc-8334) and anti-β-tubulin (1∶1000; Sigma-Aldrich, Milan, Italy) antibodies.

Band intensity was estimated using VisionWorksLS software (version 6.8, UVP, LLC). In order to detect if there were statistically significant differences among the seven distinct vectors tested, we performed a one-way ANOVA and Tukey multiple comparison test as a Post-ANOVA procedure.

### Electroretinogram Recordings

Electrophysiological recordings in mice were performed as detailed [31].

### Anti-CAR Immunostaining

Retinal cryosections were washed once in PBS and then fixed for 10 min in 4% PFA at room temperature (RT). They were then permeabilized for 1 hour in PBS containing 0.1% Triton® X-100. Blocking solution containing 10% normal goat serum (Sigma–Aldrich, St. Louis, MO) was also applied for 1 hour. The primary antibody rabbit anti-hCAR (1∶10000, kindly provided by Cheryl M. Craft, University of Southern California, Los Angeles, CA [32]) was diluted in PBS and incubated overnight at 4°C. The secondary antibody (Alexa Fluor® 594, anti-rabbit, 1∶1000; Molecular Probes, Invitrogen, Carlsbad, CA) was incubated for 1 hour at RT. Vectashield (Vector Lab Inc., Peterborough, UK) was used to visualize nuclei. Sections were photographed using a Laser Scanning Microscope LSM710 v.5.0 SP1.1, Zen 2008 Software (Carl Zeiss, Microimaging, Germany). Statistical differences in cone transduction between the various AAV serotypes were analyzed by deviance from a negative binomial generalized linear model (GLM).

## Results

### Isolation of Novel AAV Sequences from Porcine Tissues and Generation of the Corresponding AAV Vectors

AAV *rep* sequences were PCR-amplified from porcine gut genomic DNA and sequenced to identify novel isolates. Samples containing unique *rep* sequences were used as templates for subsequent nested thermal asymmetric interlaced PCR amplification of full-length *cap* sequences. Using this method, *cap* sequences were obtained for AAVpo4, -po5, and -po6 (GenBank accession: JX896667, JX896666, and JX896664, respectively), named after the order in which they were identified. Previously, only a partial sequence for AAVpo2 was isolated. Since AAV2/po2 shares high homology with AAVpo6, we were able to create a hybrid, which we called AAVpo2.1, using the beginning portion of AAVpo6 to complete the *cap* for AAVpo2 (GenBank accession JX896665). Once the full-length AAV *cap* was obtained from pig genomic DNA, the *cap* was cloned into p600 using SwaI and NotI restriction sites [25], thus generating pACK2/po1, 2/po2.1, 2/po4, 2/po5, and 2/po6. These plasmids were then used for the production of high-titer AAV vector preparations, as described in the Materials and Methods section.

**Figure 2:**
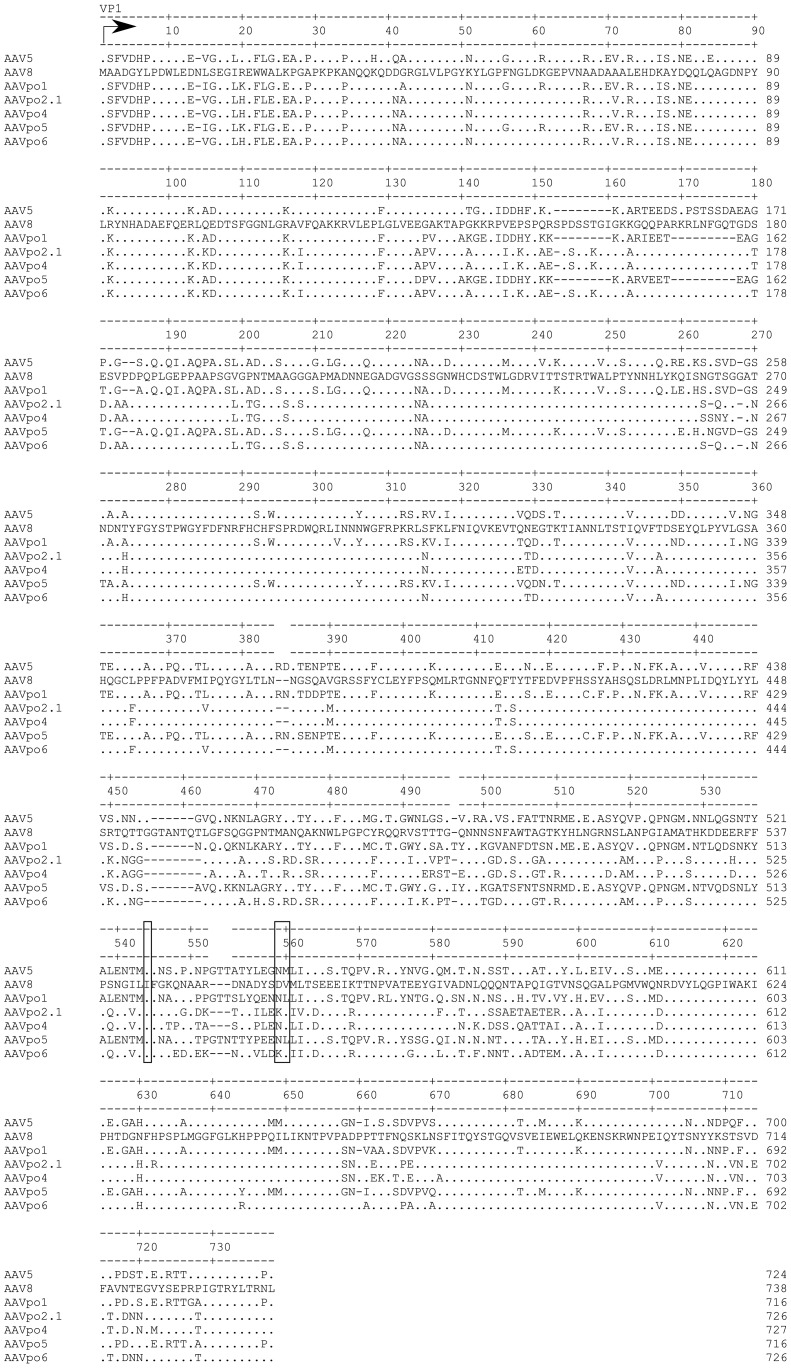
Amino acid (aa) sequence alignment of AAV5, −8, -po1, -po2.1, -po4, -po5, and -po6. Dots indicate conserved residues with AAV8, while dashes represent aa missing at those positions. The clear boxes are putative sialic acid binding domains of AAV5.

The phylogenetic relationships obtained through the alignment of the *cap* sequences of the newly isolated porcine serotypes and other preexisting AAVs are shown in Figure 1. Porcine-derived AAVs are highly divergent. AAVpo1 and AAVpo5 result to be phylogenetic neighbors and to be closely related to AAV5, a proved efficient PR transducer [19]–[21], [23]. AAVpo2.1, po6 and po4 form a separate clade. The alignment of the *cap* amino-acid sequences from AAV5, −8, -po1, -po2.1, -po4, -po5 and -po6 is shown in Figure 2.

### AAV Vectors-Mediated Transduction of Murine and Pig Retina

AAV2/po1, 2/po2.1, 2/po4, 2/po5 and 2/po6 vectors expressing EGFP under the transcriptional control of the cytomegalovirus (CMV) promoter were injected either subretinally or intravitreally in adult C57Bl/6 mice (2E9 genome copies-GC- or 5E9 GC/eye of each vector). The same doses of AAV2/5- and 2/8-CMV-EGFP were injected for comparison purposes. Animals were sacrificed one month after vector administration and retinal cryosections were analyzed under a fluorescent microscope.

**Figure 3:**
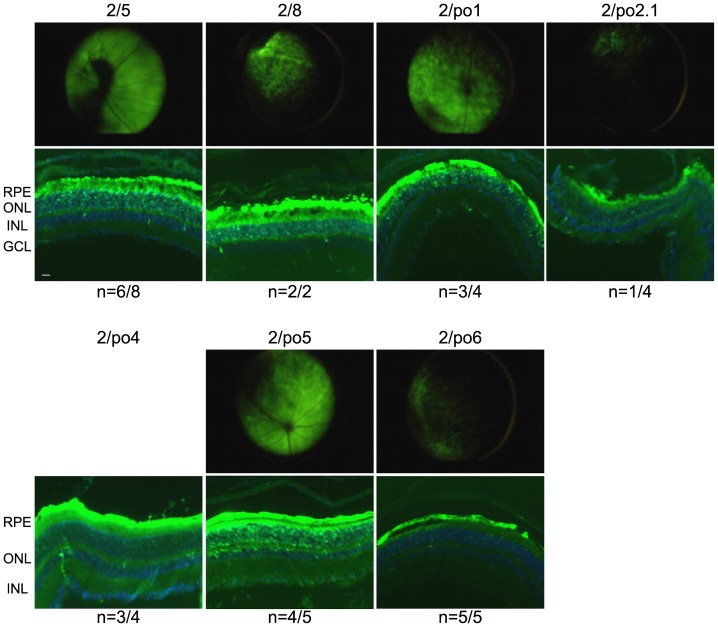
Murine retinal transduction following AAV subretinal delivery. AAV vectors (2E9 GC/eye with the exception of AAV/po1 which was injected at 5E9 GC/eye) were delivered subretinally in adult C57Bl/6 mice. One month later non-invasive fundus photographs (upper panels; photograph not available for AAV2/po4) were taken to monitor fluorescence before sacrifice, when fluorescence microscope analysis of histological sections (lower panels) was performed. n indicates the number of retinal sections resembling the representative picture out of the total number of injected retinas. The AAV vector serotypes used are indicated above each panel. RPE: retinal pigment epithelium; ONL: outer nuclear layer; INL: inner nuclear layer; GCL: ganglion cell layer. Magnification of histological sections  = 20×; scale bar  = 20 µm; exposure  = 1 sec.

Following subretinal administration, all porcine AAV serotypes efficiently transduced the RPE. PR were also transduced at variable levels, along with other cells in the inner nuclear layer (Fig. 3). Although microscopy fluorescence images of retinal cryosections are not quantitative, AAV2/po1 and 2/po5 appear to be the most efficient porcine serotypes for PR transduction. No significant or consistent inner or outer retina transduction was observed following intravitreal injection of any of the porcine AAV vectors (data not shown).

**Figure 4:**
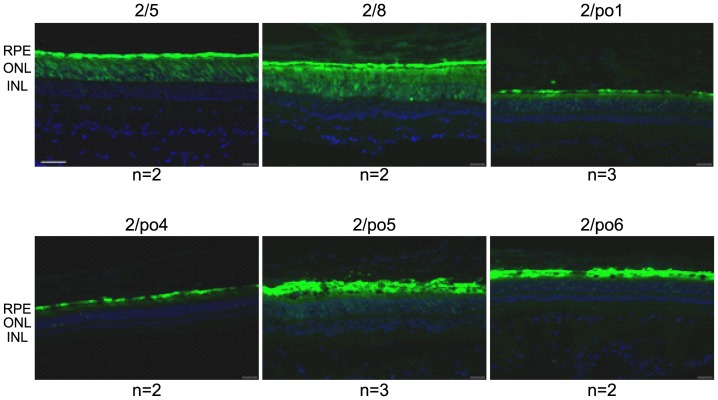
Porcine retinal transduction following AAV subretinal delivery. AAV vectors were delivered subretinally in pigs at a dose of 1E10 GC/eye. One month later eyes were harvested and retinal histological sections were analyzed by fluorescence microscopy. The n of retinas analyzed is indicated at the bottom of each panel. The AAV vector serotypes used are indicated above each panel. RPE: retinal pigment epithelium; ONL: outer nuclear layer; INL: inner nuclear layer. Magnification  = 20×; scale bar  = 50 µm; exposure  = 2 sec.

In order to analyze the pattern of transduction in a retina closer to the human than the murine in terms of evolutionary distance, size and organization, AAV vectors were injected subretinally in 11-week-old Large White pigs (1E10 GC/eye). Due to its low levels of murine outer retina transduction, AAV2/po2.1 was not tested in the porcine retina. Pig eyes were harvested one month after vector administration and fixed eyecups were dissected under a fluorescence microscope to identify the transduced area. Fluorescence microscope analysis of retinal cryosections revealed that, similar to what occurred in mice, porcine AAV serotypes transduce both RPE and PR (Fig. 4 and Figure S1). AAV2/po1 and 2/po5 are the porcine serotypes that drive the most robust expression in PR; however, the EGFP expression levels mediated by these vectors in the outer nuclear layer (ONL) appear lower than those observed with AAV2/5 and 2/8.

### AAV2/po1 and 2/po5 Transduce Murine PR Similarly to AAV2/5 and 2/8

**Figure 5:**
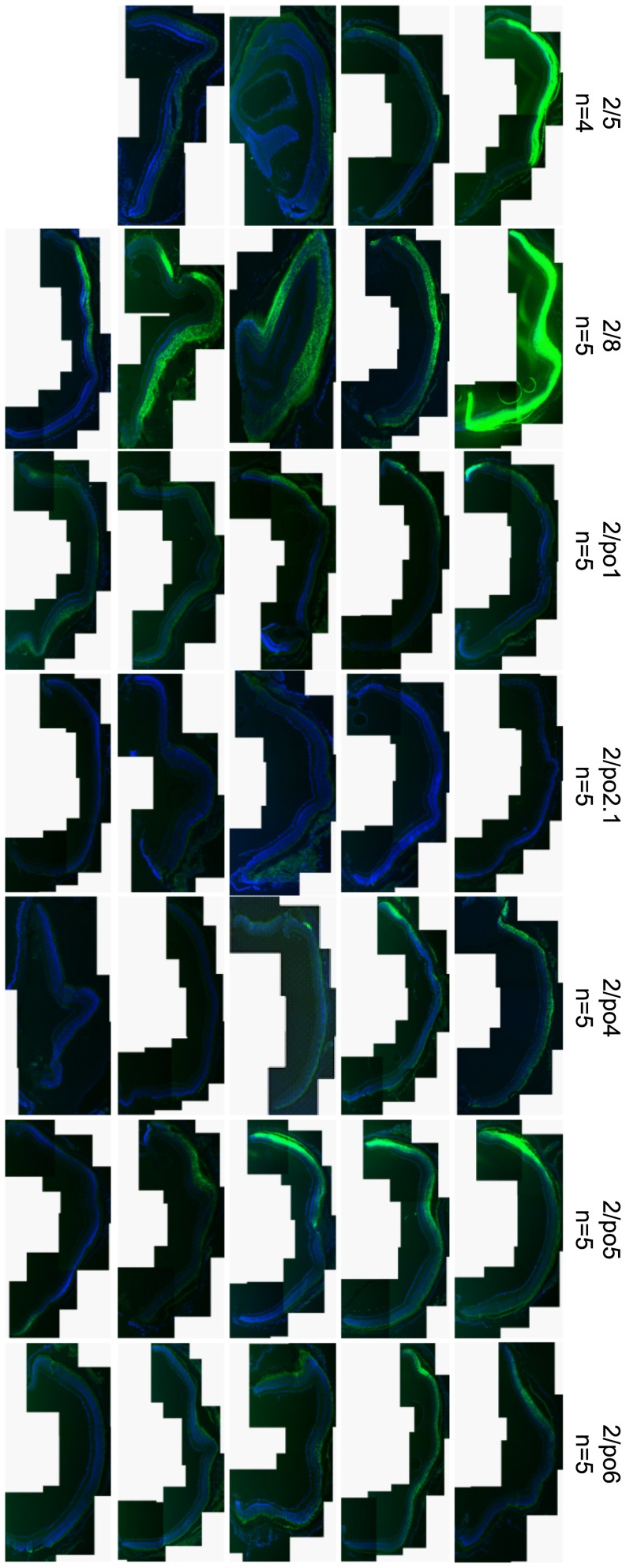
Murine PR transduction mediated by AAV vectors containing the rod-specific Rho promoter. Rho-driven EGFP fluorescence in murine retinal sections one month after subretinal delivery of AAV vectors (6E9 GC/eye). The AAV serotypes used and the n of retinas injected are indicated above each column. Magnification  = 10×; exposure  = 6 s.

To quantify AAV-mediated transduction specifically in PR, we generated vectors expressing EGFP from the human rhodopsin (Rho) photoreceptor-specific proximal promoter (U16824 GeneBank, sequence **−**800 to +6). Four-week-old C57Bl/6 mice were injected subretinally with 6E9 GC/eye of each vector. Mice were sacrificed one month later and their eyes were processed for histology (n = 4 for AAV2/5, n = 5 for the other vectors). In the histological sections shown in Fig. 5, the EGFP fluorescence observed originates from transduced PR outer segments and nuclei in the ONL. Among the retinas injected with porcine AAV serotypes, the highest levels of green fluorescence are observed in those injected with AAV2/po5. In 3 out of 5 retinas injected with AAV2/po5, the EGFP intensity is similar to that mediated by AAV2/5 and 2/8. PR transduction in the retinas injected with each of the other vectors ranked as follows: AAV2/po1, 2/po4 and 2/po6, in decreasing order. AAV2/po2.1 was shown to be the worst porcine serotype for PR transduction.

**Figure 6:**
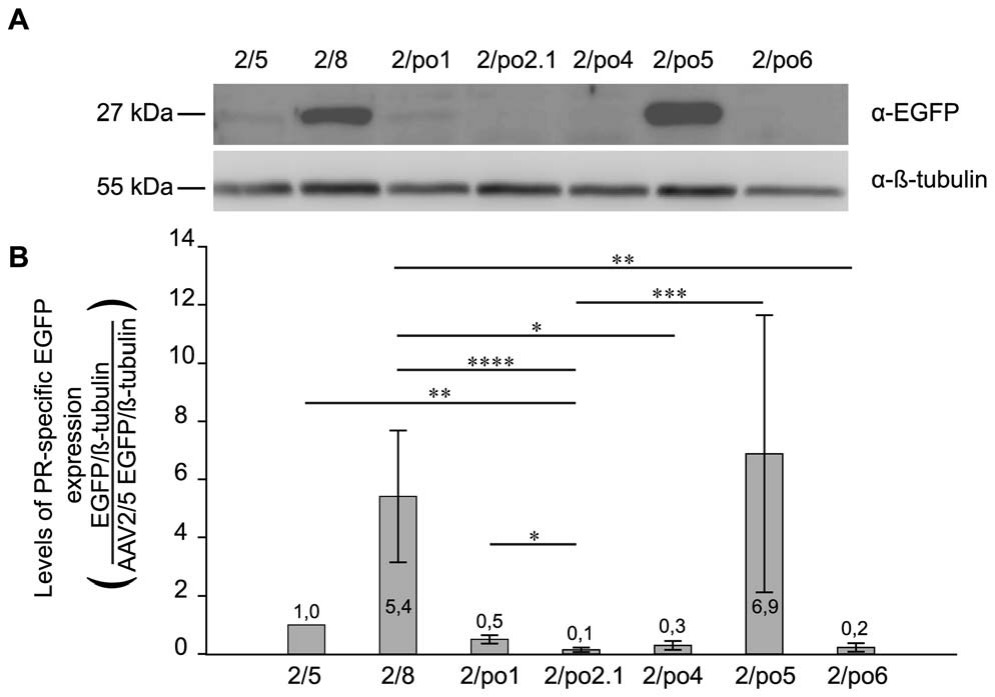
Quantification of PR-specific EGFP expression following subretinal delivery of AAV vectors. **A:** Representative Western blot out of five, each containing lysates (50 μg/lane) from retinas injected subretinally one month before with the various AAV vectors (indicated above each lane) containing the Rho promoter (6E9 GC/eye). Each blot was incubated with anti-EGFP (upper panel, α-EGFP) and anti-β-tubulin (lower panel, for normalization, α-β-tubulin) antibodies. The molecular weight is indicated on the left. **B:** Quantification of PR-specific EGFP expression mediated by each AAV vector (indicated below each bar), compared to AAV2/5. The intensity of the EGFP and β-tubulin bands was quantified and the resulting EGFP/β-tubulin ratio values were divided by those of AAV2/5. Averages (indicated above or inside each bar) ± SE for the 5 retinas injected with each AAV have been plotted. Statistical differences were calculated using one-way ANOVA (p-value  = 1,87E-5). * corresponds to p≤0.05, ** to p≤0.01, *** to p≤0.001 and **** to p≤0.0001.

To confirm this, we quantified EGFP expression by Western blot analysis of lysates of whole retinas injected one month before, as shown in Fig. 5. One representative Western blot out of the five performed is shown in Fig. 6A. The intensity of the bands (n = 5 eyes analyzed/group) corresponding to EGFP and to β-tubulin, the latter used for normalization, was quantified with an imaging software (see Materials and Methods for details). The EGFP/β-tubulin ratio values for each serotype were divided by those of AAV2/5, and the corresponding values are shown as average ± standard error (SE) in Fig. 6B. EGFP expression obtained with AAV2/po2.1 was significantly lower than that mediated by 2/5, 2/8, 2/po1 and 2/po5 (Fig. 6B). Also, 2/po4 and 2/po6 performed significantly worse than 2/8. Even if AAV2/po5 has a tendency to transduce murine PR more efficiently than the other serotypes, there were no statistically significant differences among AAV2/5, 2/8, 2/po1 and 2/po5 performances, which shows they transduce murine PR in a similar way.

### AAV2/po1 and 2/po5 Transduce Pig Cone PR Similarly to AAV2/5 or 2/8

**Figure 7:**
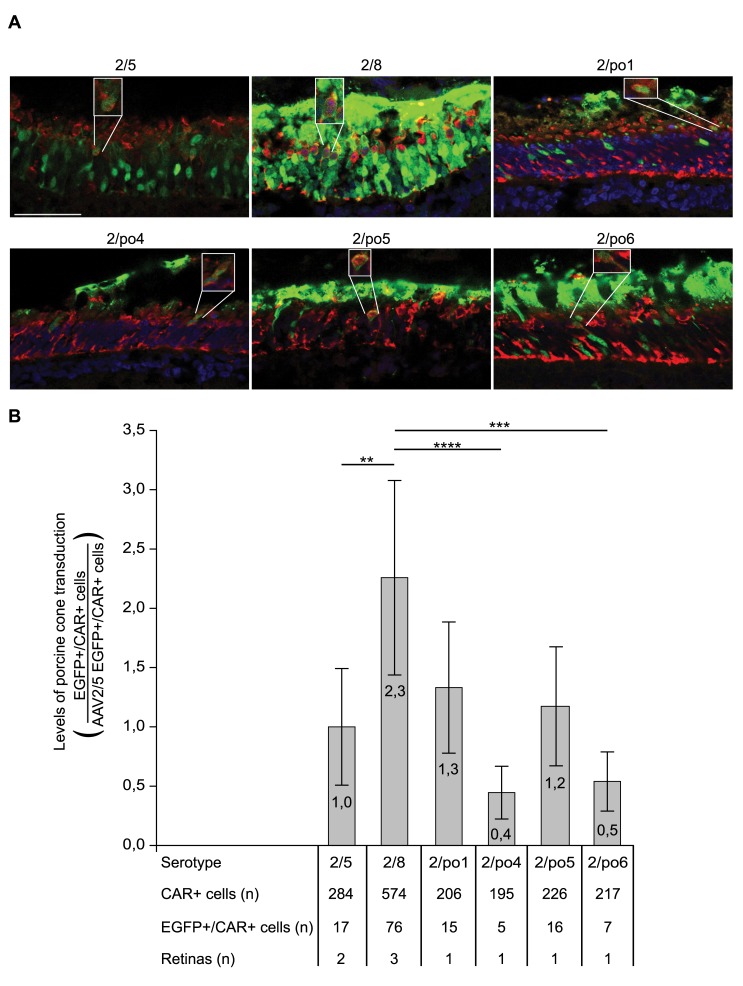
Pig cone transduction following subretinal administration of AAV vectors. **A:** Pig retinal cryosections were immunolabelled with the cone-specific anti-cone arrestin (CAR) antibody one month after subretinal delivery of AAV vectors (indicated above each picture), as in Fig. 2. Double-labelled transduced cones (EGFP+/CAR+) are shown in the insets. Confocal microscope magnification  = 63×; scale bar  = 50 µm. **B:** Quantification of cone transduction mediated by porcine AAV serotypes. The number of EGFP+/CAR+ cells obtained with each serotype was divided by the number of EGFP+/CAR+ cells obtained with AAV2/5. Values are shown as average (indicated inside each bar) ± SE. Statistical differences were calculated using GLM (p-value  = 8.6E-7). ** corresponds to p≤0.01, *** to p≤0.001 and **** to p≤0.0001.

Cone PR function is necessary for visual acuity and colour vision [33]. In IRDs, cones may degenerate either primarily or secondarily to loss of rod PR [34]. Thus, we selected pigs rather than mice to study cone PR transduction levels mediated by the various AAV serotypes. The pig retina, which contains a streak region enriched in cones and has an overall high cone:rod ratio [26], is ideal for studying cone transduction mediated by viral vectors. Cone PR were visualized by immunolabelling (with an anti-cone arrestin -CAR- antibody) retinal sections from porcine eyes that were injected with AAV vectors, as shown in Fig. 4 (and Fig. S1). For each AAV vector, a minimum of 195 CAR-positive cells were analyzed under a laser scanning microscope for any additional expression of EGFP (Fig. 7A). Cells expressing both EGFP and CAR were considered transduced cone PR (Fig. 7A insets). The mean values of cones transduced by each AAV vector were divided by the mean values of those transduced by AAV2/5 (Fig. 7B). AAV2/8 has cone transduction levels that are significantly higher than AAV2/5, 2/po4 and 2/po6, but that are not higher than 2/po1 and 2/po5. This suggests that 2/po1 and 2/po5 are the most efficient porcine serotypes for pig cone PR transduction among those tested.

### Porcine Retinal Structure and Function are Preserved after Subretinal Delivery of Porcine AAVs

**Figure 8:**
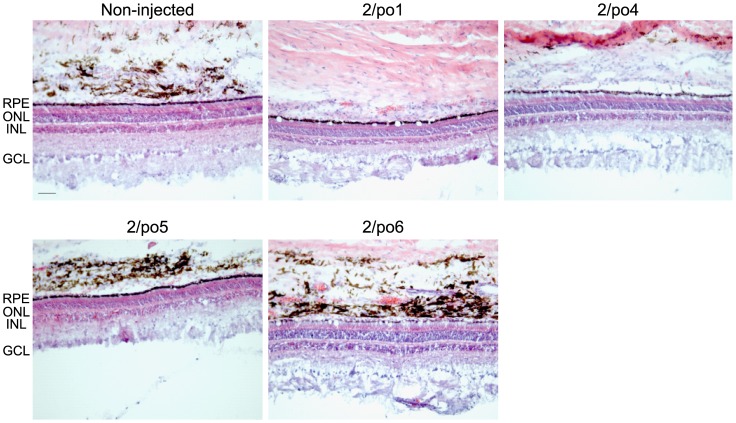
Subretinal delivery of AAV vectors does not alter porcine retinal histology. Hematoxylin and eosin staining of pig retina cryosections one month after transduction with 1E10 GC/eye of each AAV vector (indicated above each picture), as in Fig. 2. Non-injected retinas were used as controls. RPE: retinal pigment epithelium; ONL: outer nuclear layer; INL: inner nuclear layer; GCL: ganglion cell layer. Magnification  = 20×; scale bar  = 50 µm.

Pig retina architecture was analyzed following hematoxylin and eosin staining of EGFP-positive pig retinal cryosections from animals treated one month before with subretinal injections of AAV vectors, as shown in Fig. 4. No cellular infiltrates, malformations or reduction of the ONL thickness were appreciated (Fig. 8).

In addition, murine retinal function was assessed one month after vector delivery by Ganzfeld electroretinograms (ERG) in both scotopic and photopic conditions. No statistically significant differences were found between retinas that were not injected with AAV vectors (n = 3) and those that were (n = 5 for each serotype). The maximum a-wave and b-wave averages for each serotype are shown in Table 1. This indicates that retinal function is preserved after porcine AAV administration.

**Table 1:** Ganzfeld electroretinogram (ERG) maximum amplitude values in retinas non-injected or injected with AAV2/5, 2/8 and porcine AAV vectors.

*Maximum Amplitude Mean µV (±SE)*	*Non-Injected*	*AAV2/5*	*AAV2/8*	*AAV2/po1*	*AAV2/po2.1*	*AAV2/po4*	*AAV2/po5*	*AAV2/po6*
*a-wave*	350.1 (±41.4)	303.6 (±13)	340.8 (±15.8)	294.1 (±38.1)	353.5 (±53)	312.5 (±13.7)	357.2 (±24.3)	328.3 (±22.6)
*b-wave*	691.7 (±38.5)	606.4 (±24.7)	654.3 (±36.9)	547.9 (±72.4)	635.6 (±99.3)	610.1 (±39.5)	647.1 (±30.2)	601.4 (±80.5)

NB: Values in the table are the average maximum a-waves and b-waves amplitudes obtained by ERG under scotopic and photopic conditions. No statistically significant differences were found between values of non-injected (n = 3) retinas and injected with AAV vectors (n = 5 for each serotype). SE: standard error.

## Discussion

Efficient PR gene transfer is essential for successful gene therapy of IRDs, most of which are due to mutations in genes expressed in PR. AAV2/5 and 2/8 provide robust levels of PR transduction across various species, including NHP [19]–[23], [35]. This has allowed us to perform effective gene therapy in some IRD animal models [35]–[43], but not in others [31]. However, because high levels of widespread PR transduction are required to treat the most severe forms of IRDs including LCA, the search for AAV serotypes with more robust PR transduction characteristics than AAV2/5 and 2/8 is ongoing.

To achieve this, several groups have mutagenized capsids of naturally-occurring serotypes either with systematic, unbiased approaches [44], or by changing specific amino-acid residues based on their predicted role in AAV-mediated transduction [45]. Although successful in some instances [46], these approaches are laborious and often result in the development of AAV vectors with low production yields/transduction levels compared to the parental serotype. Tissues from mammals that are AAV natural hosts provide a theoretically endless source of AAV sequences, which can easily be amplified and isolated for the generation of recombinant vectors. These vectors, in turn, can be produced at high titers and are highly infectious. Indeed, capsid sequences from dozens of naturally-occurring AAVs have been isolated thus far from several mammalian tissues, including human and non-human primates [47]–[48], providing some of the most effective gene therapy vehicles for transduction of primary targets, such as liver [49] or retina [22]. Here we set about to test the retinal transduction characteristics of five AAV vectors containing capsids whose sequences have been isolated from porcine tissues. All but AAV2/po1 [25] had not been described before.

We previously reported that AAV2/po1 transduces murine PR similarly to AAV2/5, based on a highly divergent serotype which is phylogenetically close to 2/po1 [25]. Our current results confirmed this quantitatively using the PR-specific Rho promoter. AAV2/po5, whose *cap* sequence is phylogenetically close to both AAV5 and -po1 (Fig. 1), also showed similar levels of murine PR transduction. It is possible that vectors which are phylogenetically close are comparable in their ability to transduce PR. For instance, they could bind similar receptors. α-2,3-*N*-linked sialic acid has been reported to bind to AAV5 [50]–[51]. Based on the sialic acid binding residues of the canine parvovirus (CPV) capsid, it has been predicted that the sialic acid binding residues on the surface of the AAV5 capsid are I528, N546, and M547 [52]. The aminoacidic sequence alignment of AAV5, -po1, and -po5 shows that I528 and N546 are conserved (Fig. 2).

AAV2/8 is considered the current gold standard for PR transduction across several species [19]-[20], [22], [53]. While AAV2/8 outperformed AAV2/po2.1, 2/po4 and 2/po6, its levels of murine PR transduction were not statistically different from those achieved with AAV2/po1 and AAV2/po5, confirming that the latter are efficient vehicles for PR targeting.

The ability of porcine AAV vectors to transduce the retina and, more specifically, PR, was then confirmed in pigs. Indeed, the porcine eye shares several anatomic and physiological features with the human eye, and their dimensions are similar [54]. The retina of the pig also resembles the human retina in that it has an extensive vascular tree fed by four large retinal arteries, and although there is no true fovea, the porcine retina has a streak area that contains PR with a high cone to rod ratio [26]. Moreover, the distribution of immunocompetent cells in the porcine retina largely resembles that observed in the human retina [55]. Therefore, the transduction pattern observed in the pig retina is likely to reflect the pattern one would expect to find with the same vectors in the human retina. An additional reason to test vectors for retinal gene therapy in pigs is that several pig models and genetic mutants for ocular diseases exist [56]–[58]. We found that even if the four porcine AAV serotypes transduce the pig ONL less efficiently than AAV2/8 and 2/5, there is no statistical difference regarding cone transduction mediated by AAV2/5, 2/8, 2/po1 and 2/po5, suggesting that the latter are the most efficient porcine vectors for cone transduction.

Overall, we have shown that the porcine AAV serotypes AAV2/po1 and 2/po5 transduce RPE and PR of mice and pigs at levels similar, though not superior, to those obtained using AAV2/5 and AAV2/8, and should therefore be considered for retinal gene therapy along with more studied serotypes.

## Supporting Information

Figure S1 **Porcine retinal transduction following AAV subretinal delivery.** AAV vectors containing the CMV-EGFP expression cassette were subretinally injected in Large White female pigs (dose of 1E10 GC/eye). Vectors were delivered in the avascular nasal area of the posterior pole between the two main vascular arches (**Ai**) which is a region with high cone density [26]. One month later eyes were harvested and transduced regions were sampled under a fluorescence stereomicroscope (**Aii**) for further cryosectioning. (**B**) Histological images from porcine retinas injected with AAV2/5, 2/8, 2/po1, 2/po4, 2/po5 and 2/po6 (as indicated in **Aii,** n = 1 for all serotypes except n  = 2 for AAV2/8) were analyzed by fluorescence microscopy. Each of these is a montage of 10× single photographs. Arrowheads delimitate the area with transduced PR. Magnification  = 10×; scale bar  = 500 µm; exposure  = 6 sec.
(TIF)
