# Supplementary figures and images for: Recombinant Vectors Based on Porcine Adeno-Associated Viral Serotypes Transduce the Murine and Pig Retina
Source: PLoS One. 2013 Mar 8;8(3):e59025. doi: 10.1371/journal.pone.0059025 (PMC3592811; doi:10.1371/journal.pone.0059025)

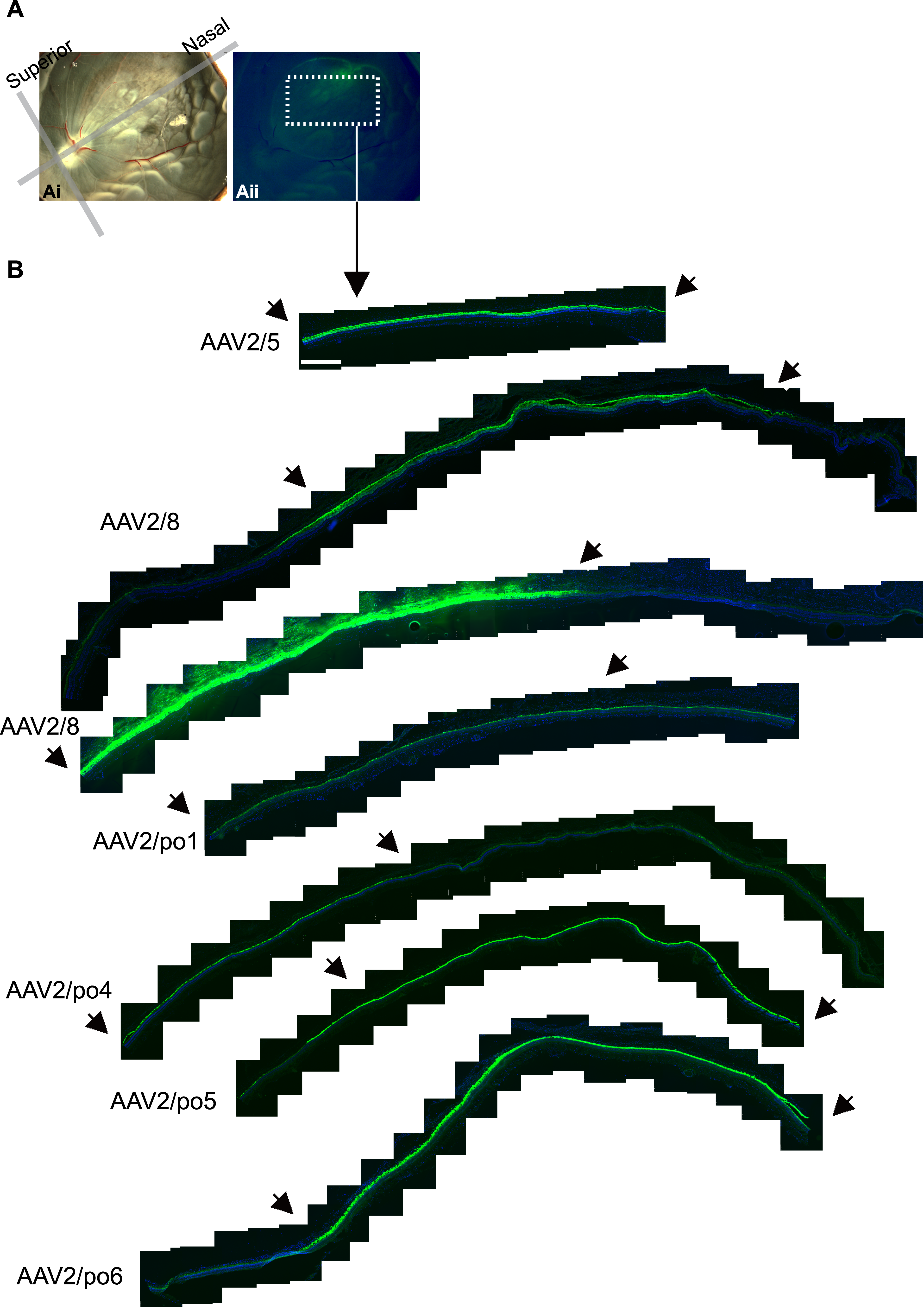

Supplement: Figure S1 — Porcine retinal transduction following AAV subretinal delivery. AAV vectors containing the CMV-EGFP expression cassette were subretinally injected in Large White female pigs (dose of 1E10 GC/eye). Vectors were delivered in the avascular nasal area of the posterior pole between the two main vascular arches (Ai) which is a region with high cone density [26]. One month later eyes were harvested and transduced regions were sampled under a fluorescence stereomicroscope (Aii) for further cryosectioning. (B) Histological images from porcine retinas injected with AAV2/5, 2/8, 2/po1, 2/po4, 2/po5 and 2/po6 (as indicated in Aii, n = 1 for all serotypes except n = 2 for AAV2/8) were analyzed by fluorescence microscopy. Each of these is a montage of 10× single photographs. Arrowheads delimitate the area with transduced PR. Magnification = 10×; scale bar = 500 µm; exposure = 6 sec. (TIF) [file pone.0059025.s001.tif]
